# Guarani Virophage, a New Sputnik-Like Isolate From a Brazilian Lake

**DOI:** 10.3389/fmicb.2019.01003

**Published:** 2019-05-03

**Authors:** Said Mougari, Meriem Bekliz, Jonatas Abrahao, Fabrizio Di Pinto, Anthony Levasseur, Bernard La Scola

**Affiliations:** ^1^MEPHI, APHM, IRD 198, Department of Medicine, IHU-Méditerranée Infection, Aix-Marseille University, Marseille, France; ^2^Laboratório de Vírus, Instituto de Ciências Biológicas, Departamento de Microbiologia, Universidade Federal de Minas Gerais, Belo Horizonte, Brazil

**Keywords:** Guarani virophage, mimiviruses, large host specificity, late replication, mimivirus replicative cycle, amoeba co-culture

## Abstract

Virophages are critical regulators of viral population dynamics and potential actors in the stability of the microbial networks. These small biological entities predate the replicative cycle of giant viruses, such as the members of the *Mimiviridae* family or their distant relatives, which produce within the cytoplasm of their host cells a viral factory harboring a complex biochemistry propitious to the growth of the smaller parasites. In this paper, we describe the isolation and the characterization of a new virophage, the eighth, that we named Guarani. We observed that Guarani exhibits a late replication cycle compared to its giant virus host. In addition, like all Sputnik strains, Guarani is able to infect the three lineages A, B and C of the *Mimiviridae* family, and affects the replication and the infectivity of its host virus. In terms of genetic content, Guarani has a 18,967 bp long double-stranded DNA genome encoding 22 predicted genes very similar to Sputnik genes, except for ORF19 and ORF12. The former is more related to Zamilon while the latter seems to be novel. The architecture of the Guarani genome is closely related to Sputnik and Zamilon strains, suggesting a common origin for all these virophages.

## Introduction

The discovery of Acanthamoeba polyphaga mimivirus (APMV) in 2003 challenged the current definition of a virus. With a capsid size of 500 nm and a double-stranded DNA genome of 1.2 megabase pairs, APMV surpasses some unicellular organisms in terms of morphological dimensions and genetic content (La Scola et al., 2003; Raoult et al., 2004). In 2005, APMV became the founding member of the family *Mimiviridae*, a new taxonomic group created to allocate its outstanding features (La Scola et al., 2005). Since then, several new mimivirus strains have been isolated and classified by phylogenomics into three lineages that have been named, A, B and C. APMV is the pioneering member of the lineage A, while Moumouvirus and Megavirus chiliensis are the prototypes of the lineages B and C, respectively (Arslan et al., 2011; Yoosuf et al., 2012).

Over the last decade, the study of mimiviruses paved the way for the isolation of a remarkable new class of parasitic agents that depend on mimiviruses presence to replicate in their host cells. This new viral entity was named virophage according to its typical reproduction strategy (La Scola et al., 2008). Virophages are small viruses with icosahedral capsids ranging from 50–74 nm and 17–29 kb pairs double-stranded DNA genomes. They encode approximately 20 putative genes which expression seems to be governed by the transcription apparatus of the host mimivirus. Additionally, some virophage infections appear to decrease the infectivity and increase the production of deformed particles of their host viruses (Bekliz et al., 2016). Sputnik is the first biological entity that was consistent with this definition. It was discovered in association with Acanthamoeba castellanii mamavirus, a mimivirus from lineage A, isolated in water from a cooling tower in Paris, France. Sputnik reproduction impaired the viral cycle, and was associated with abnormal morphogenesis of mamavirus virions, with a significant decrease in the viral burst sizes (La Scola et al., 2008). Subsequently, other strains of Sputnik were isolated. Sputnik 2 was isolated with Lentille virus from the contact lens fluid of a patient with keratitis (Desnues et al., 2012). Rio negro virophage (RNV), a Sputnik 2-like strain, was isolated in association with Sambavirus from the Negro river, Brazilian Amazon (Campos et al., 2014). Finally, Sputnik 3 was found free of host virus, in a soil sample collected from Marseille, France (Gaia et al., 2013). Additionally, Mavirus, the first marine virophage, was discovered with Cafeteria roenbergensis virus, a relatively distant mimivirus, of the *Cafeteriavirus* genus, which infects the marine zooplankton *C. roenbergensis* (Fischer and Suttle, 2011).

Recently, a new divergent virophage, named Zamilon, has been isolated in association with the Mont1 virus, a mimivirus belonging to the lineage C. Unlike Sputnik, which is capable of infecting all three mimivirus lineages, Zamilon is the only virophage that can infect both B and C mimiviruses, but not lineage A (Gaia et al., 2014). This host specificity of Zamilon is explained by the presence of the Mimivirus virophage resistance element known as the MIMIVIRE system, which seems to confer to lineage A mimiviruses a nucleic-acid-based immunity against Zamilon infection (Levasseur et al., 2016b).

More recently, another virophage of 50 nm in diameter invading the viral factory of KSL5 virus, a Mimivirus-like giant virus, has been observed within *Saccamoeba* sp. inoculated with an environmental sample (Michel et al., 2018).

In parallel with the subsequent isolation allowed by co-culture strategies, 57 complete and partial virophage genomes were assembled by metagenomics from distinct environmental settings in North America, Asia and Antarctica, revealing the wide distribution of virophages (Yau et al., 2011; Santini et al., 2013; Zhou et al., 2013, 2015; Bekliz et al., 2015; Yutin et al., 2015; Gong et al., 2016; Oh et al., 2016; Roux et al., 2017).

In this paper, we describe the isolation and the characterization of a new virophage, that was formerly detected by PCR in a water sample collected from Pampulha lagoon, Belo Horizonte, Brazil. Because of the absence of an associated host giant virus in the sample, we took advantage of the co-culture strategy previously used by Gaia et al. (2013), which is based on the use of a helper giant virus, to cultivate the virophage. Transmission electron microscopy and real-time PCR were then used to explore the replicative cycle, the impact on the host giant virus, as well as the multiplication spectrum of Guarani virophage among the three lineages of the *Mimiviridae* family.

## Materials and Methods

### Molecular Screening of the Environmental Sample for the Presence of Virophages and Mimiviruses

The molecular detection of virophage and mimivirus signatures was performed on a water sample collected in Pampulha lagoon, Belo Horizonte, Brazil. The DNA was extracted as previously described by Gaia et al. (2013). For virophage detection, we used primer pairs targeting Major capsid protein encoding genes (MCPs) of Sputnik and Zamilon (V20 and gp06, respectively). A combination of primer pairs targeting polB genes, representative of the three lineages of the *Mimiviridae* family, was used for the detection of giant virus (Table 1). Real-time PCRs was performed as described below.

**Table 1:** Primers used for the environmental detection of giant viruses and virophages.

Primers	Sequences	Tm	Target
Fw-Mimi (A)	5′- GAAAATGGTGAAGAGAAAACTGA -3′	55°C	PolB gene
Rv-Mimi (A)	5′- ACCAGGATAAATGGATGCAA -3′	55°C	PolB gene
Fw-Moumou (B)	5′- AAGGGGACAAGGAGTTAAAATAT -3′	50°C	PolB gene
Rv-Moumou (B)	5′- TAGATATACGTTTGGTTTTGGAGTGA -3′	50°C	PolB gene
Fw-Mega (C)	5′- AGTTACCCAACCACAAGAAGA -3′	50°C	PolB gene
Rv-Mega (C)	5′- CAGAAGGACTAACAAAAGAACCA -3′	50°C	PolB gene
Fw-Sputnik	5′- GAGATGCTGATGGAGCCAAT -3′	59°C	MCP gene
Rv-Sputnik	5′- CATCCCACAAGAAAGGAGGA -3′	59°C	MCP gene
Fw-Zamilon	5′- GGGATGAACATCAAGCTGGT -3′	59°C	MCP gene
Rv-Zamilon	5′- GGGTTGTTGGAAGCTGACAT -3′	59°C	MCP gene

### Guarani Virophage Isolation

All PCR systems targeting the three *Mimiviridae* lineages as well as the Zamilon sequence were negative. Our PCR system targeting the Sputnik-like signature provided a positive result. Therefore, we tried to inoculate the sample in co-culture with *Acanthamoeba castellanii* and *Acanthamoeba polyphaga* cells, in order to investigate the presence of giant viruses that were not detectable in our PCR systems. However, all our efforts to cultivate a potential giant virus associated with the virophage failed in this study. Consequently, we adopted the same co-culture strategy described by Gaia et al. (2003), which is based on the use of a reporter giant virus. After several daily subcultures of the sample using APMV and *Acanthamoeba castellanii* as support, we were able to detect a constant increase in the virophage DNA amounts after each passage. This suggests that the virophage successfully replicates with the APMV and that this mimivirus can be used to produce the virophage.

### Guarani Virophage Production

Acanthamoeba polyphaga mimivirus was used to produce Guarani in co-culture with *A. castellanii* within PYG (Peptone – Yeast extract – Glucose) medium. After the lysis, the co-culture was centrifuged at 10 000 g for 10 min to pellet the mimivirus particles and the residual amoebas, and then the obtained supernatant was successively filtered through 0.8-, 0.45-, and 0.22- μm pore filter. In the last step, Guarani particles was concentrated by ultracentrifugation (60 000 g for 2 h). Then, we resuspended the pellet with PAS (Page’s Amoeba Saline) medium and submitted it subsequently to a final round of ultracentrifugation across a 15% sucrose layer to obtain a pure highly concentrated virophage suspension. The virophage suspension was stored at −80°C. The absence of giant virus particles was confirmed by negative staining electron microscopy.

### Giant Virus Production

Giant viruses used in this study were produced in PYG medium by inoculation of fresh *A. castellanii* at a suspension of 5.10^5^ cell/ml with each virus strain at a M.O.I. (Multiplicity of infection) of 10 TCID_50_. For Faustovirus, *Vermamoeba vermiformis* was used as host cell. After complete lysis of the cells, each culture was centrifuged at 1000 g for 10 min, and then the supernatant was filtered through a 0.8 μm membrane to remove residual amoebas and cysts (the filtration was performed only for Mimivirus, Faustovirus and Marseillevirus). Next, the supernatant obtained from the first step was submitted to three cycles of wash with Page’s modified Neff’s amoeba saline (PAS) by ultracentrifugation for 1 h (14 000 g for mimiviruses, Pandoraviruses, Pithovirus, Cedravirus. 40 000 g for Faustovirus and Marseillevirus).

### Host Range Studies

A collection of several mimivirus strains representative from the three lineages including, APMV from group A, Moumouvirus from group B and Megavirus Courdo-11 from group C (La Scola et al., 2003, 2010), was used to test the host spectrum of Guarani virophage. In addition, the virophage was tested against other Megavirales members including Pandoravirus massiliensis and Pandoravirus braziliensis (Aherfi et al., 2018), Pithovirus massiliensis (Levasseur et al., 2016a), Cedravirus A11 (Andreani et al., 2016), Faustovirus E12 and Marseillevirus (Yutin et al., 2009; Rossmann et al., 2015). A suspension containing fresh *A. castellanii* cells, the cellular support of the system, was prepared by a successive round of centrifugations (1000 g for 10 min) and resuspension in PAS medium. For Faustovirus, *Vermamoeba vermiformis* was used as host cells. Ten ml of amoeba at 5 × 10^5^ cell/ml were inoculated with each of the single mimivirus strain (MOI = 10 TCID_50_) simultaneously with Guarani virophage (MOI = 10). After 1 h of incubation at 32°C for virus-virophage adsorption, the supernatant was removed by three rounds of successive centrifugations to eliminate extracellular giant virus and Guarani particles. The cells were then resuspended in fresh PAS medium and submitted for a second incubation for 24 h at 32°C. This time point was defined as H0. As negative control, we incubated each giant virus strain separately with amoeba in absence of virophage. At time points H0 and H24 p.i., a 200 μl aliquot of each co-culture was collected for real-time PCR.

### Evaluation of Guarani Effect on the Replication, the Infectivity, the Morphogenesis and the Ability of Mimivirus to Lyse the Host Cells

To assess the impact of the virophage on the DNA replication, the morphogenesis, and the infectivity of APMV, *A. castellanii* cells were inoculated with APMV at M.O.I. = 1 TCID_50_ and the virophage at M.O.I. = 10, as described above. At times points H0, H4, H8, H12, and H16 p.i., a 1 ml aliquot of the co-culture was collected for real-time PCR and transmission electronic microscopy analysis. A 1 ml aliquot was also collected after 48 h p.i. for virus titration. The measure of the impact on Mimivirus DNA replication was performed by real-times PCR targeting PolB gene of the virus (Table 1). The effect on the infectivity was evaluated by virus titration (see below). The rate of abnormal particles was determined by analyzing the virus factories of 200 co-infected cells. In parallel, the effect of Guarani on amoeba survival was estimated by counting living trophozoites at times points H24 and H48 p.i. All the experiments were performed in triplicate using APMV infected amoebas as controls (except for the transmission electronic microscopy analysis which was performed once).

### Virus Titration

96-well plates with 4.10^4^ *A. castellanii* cells in 50 μl of PYG medium per well were used as support for the virus titration. The virus suspensions were serially diluted in 100 μl of PAS medium (from 10^−1^ to 10^−10^) and added to each well. After 5 days of incubation at 32°C, the Cytopathic effects (CPEs) were determined by observing cells under light microscope, and the titer was calculated by the Reed and Muench method (Reed and Muench, 1938).

### The Viral Fitness of Guarani

*Acanthamoeba castellanii* cells were co-infected with Mimivirus, Moumouvirus or Courdo-11 and Guarani virophages at an M.O.I of 10 for each virus. The infection was performed in triplicate as described above. At times points H0, H4, H8, H12, and H16 p.i., a 200 μl aliquot was collected. Real-time PCR was used to measure the DNA replication of the virophage using the same primers listed in Table 1 for Sputnik (ORF22 in Guarani genome).

### Real-Time PCR

DNA extraction was performed from 200 μl of each co-culture using the EZ1 DNA Tissue Kit (Qiagen, Hilden, Germany), following the manufacturer’s instructions. Real time PCRs were performed in a CFX96 thermal cycler (Bio-Rad Laboratories) using a SYBR Green PCR Master Mix (Qiagen) according to the manufacturer’s instructions. The results were analyzed by ΔCt method considering the difference between time H0 and times H4, H8, H12, H16 or H24 p.i. (according to the experiment).

### Transmission Electronic Microscopy

Co-infected cells were washed three times with PBS solution and then fixed overnight with 2% glutaraldehyde in 0.1 M cacodylate buffer. Cells were washed again with 0.1 M cacodylate buffer and 1% osmium tetroxide in 0.1 M potassium ferricyanide solution was used for second fixation of 1 h. Samples were dehydrated in increasing ethanol concentrations (from 30 to 100%) and subsequently embedded in Epon 812 resin. Sections (70 nm) were stained with 3.5% uranyl acetate and lead citrate before examination with a transmission electron microscope.

### Sequencing and Bioinformatics Analysis

Guarani genome reads obtained from MiSeq sequencer (Illumina) were trimmed using Trimmomatic (Bolger et al., 2014), then assembled into one scaffold through Spades software (default parameters) and manual finishing (Bankevich et al., 2012). Coding DNA sequences were predicted using GeneMarkS (Besemer, 2001), and functional annotation was carried out using BLASTp searches (Basic Local Alignment Search Tool) against the Non Redundant protein collection of the NCBI, COGs and NCVOGs databases (e-value < 1 × 10^−3^).

Conserved domain analysis, for both annotated genes and ORFans (ORF12 only), has been performed using CD-Search (NCBI database) and InterPro scan (EBI database) (Marchler-Bauer and Bryant, 2004; Hunter et al., 2009; Supplementary Table S1). For ORF12, protein homology detection and structure prediction were conducted using HHpred and Phyre2 (Kelley et al., 2015; Söding et al., 2005). However, theses analyses failed to provide additional information to what has been found by BLASTp search.

Protein sequence comparisons with the other Sputnik-like virophages were made using BLASTp searches against the virophage gene collection in the NCBI database. Nucleotide sequence comparisons were made using BLASTn. Gene synteny and nucleotide divergence between available virophage genomes were analyzed using the Circos software (Krzywinski et al., 2009).

### Genome Sequence Submission

Guarani genome is available in the EMBL-EBI database under accession number LS999520.

### Phylogenetic Analysis

Virophage genes were aligned using Muscle software (Edgar, 2004). Phylogenetic trees reconstruction was performed with the MEGA7 software using the maximum-likelihood algorithm and the most appropriate substitution model (Tamura et al., 2011).

### Promoter Research

The intergenic regions were manually extracted from the Guarani genome. A total of 21 sequences were found in both positive (16 intergenic regions) and negative strands (5 intergenic regions). For comparison, the length of the intergenic region in the positive strand was 3562 bp vs. 15405 bp for the coding region. The search for promoter motif was performed using the Fuzznuc program of the EMBOSS software. The late Mimivirus promoter was searched using the same motif pattern previously described for Mimivirus and Sputnik, with one mismatch allowed [AT](8)T[AC]TN(4)[AT](5)[AG]TA[TG]A (Poirot et al., 2010). The early Mimivirus promoter was searched with no mismatch using the following sequence AAAATTGA (Claverie, 2005). The Berkeley Logo platform^1^ was used to create the promoter sequence logo.

## Results

### Guarani Virophage Isolation, Description, and Replicative Cycle

**FIGURE 1:**
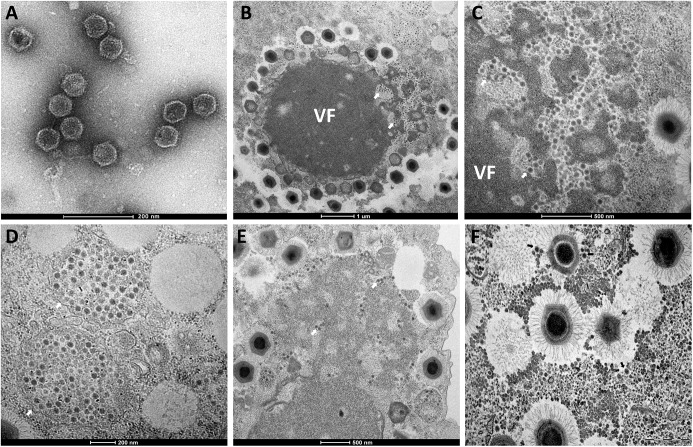
Morphological aspect and different intracellular locations of Guarani. **(A)** Observation by negative staining electron microscopy. **(B–E)** Observations by transmission electron microscopy. **(A)** Purified Guarani virophage particles (scale bar 200 nm). **(B,C)** Guarani particles infiltrating the Mimivirus factory (arrows) (scale bar 1 μm and 500 nm). **(D–F)** Cytoplasmic Guarani progeny (arrows) (scale bars 200, 500, and 500 nm, respectively).

While attempting to find new virophages of giant viruses, we carried out a molecular screening of a water sample collected from an artificial lake located in the Pampulha lagoon, Belo Horizonte, Brazil. We detected the presence of a Sputnik-like signature based on the Major capsid protein PCR targeting system. The virophage was found free of giant virus, but we were able to cultivate it in co-culture in *Acanthamoeba castellanii* by using APMV as a helper giant virus. The new isolated virophage was named Guarani, in tribute to the South American Guarani Indigenous tribes near where the virus was isolated. As demonstrated by negative staining electron microscopy of Guarani purified particles, the virophage had a typical icosahedral small particle with a diameter of 50–60 nm approximately (Figure 1A).

**FIGURE 2:**
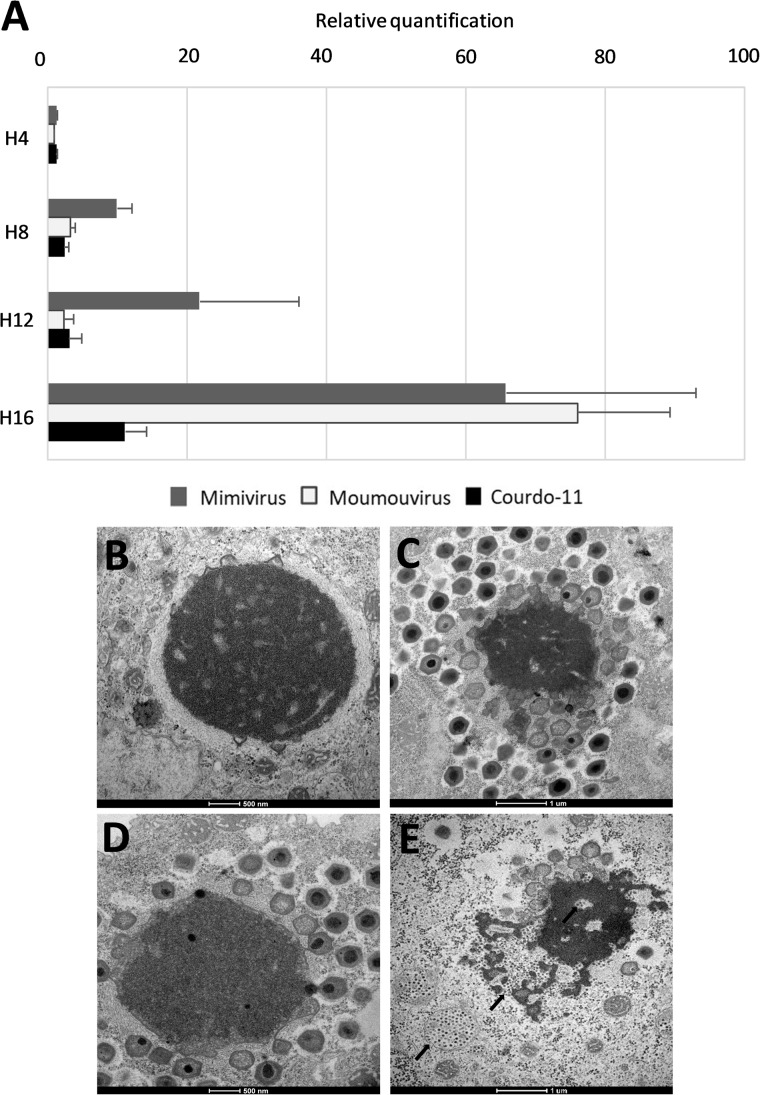
Guarani replication during coinfection. **(A)** Histogram of Guarani growth during co-infection of *Acanthamoeba castellanii* in the presence of Mimivirus, Moumouvirus or Courdo-11 as reporters. The replication was measured by Real-Time PCR from H0-to-H16 post-infection and calculated by the difference in the Cycle threshold (Ct) between time points H0 and H4, H8, H12, and H16, respectively. The results were analyzed with the Delta Ct method. **(B–E)** Transmission electron microscopy images of Mimivirus and Guarani co-infecting *A. castellanii.* **(B)** At 4 h post-infection (scale bar 500 nm). **(C)** At 8 h post-infection (scale bar 1 μm). **(D)** At 12 h post-infection (scale bar 500 nm). **(E)** Important production of Guarani particles within and around the Mimivirus factory at 16 h post-infection (arrows) (scale bar 1 μm).

In order to describe the kinetics of production of Guarani virophage with its host giant virus, as well as the specific locations of each one during the viral cycle, amoebas co-infected with APMV and Guarani were observed through transmission electron microscopy at sequential stages of co-infection. Unlike APMV particles that begin to emerge from their virus factory at H4 post-infection (p.i.), after the viral eclipse phase (Figure 2B), we failed to detect Guarani particles at H4, H8, H12 p.i. time points (Figure 2B–D). Nevertheless, at H16 p.i. we detected a high production of the virophage in the infected cells (Figure 2E). We were able to visualize Guarani particles being produced within the virus factories, which are the production sites of the host mimivirus (Figure 1B,C). Likewise, the virophage progeny were also observed in the cytoplasm of the infected cells, clustered in a typical manner (Figure 1D) or scattered around different intracellular compartments and mimivirus particles (Figure 1E,F).

Next, we used real-time PCR to measure the replication of Guarani DNA during its viral cycle. Giant viruses representative of the three lineages of the family *Mimiviridae* (Mimivirus from lineage A, Moumouvirus and Courdo 11 from lineage B and C, respectively) were used to test the replication efficiency of the virophage. Our data strongly suggests that Guarani genome replication starts at a late stage (at H4 post co-infection approximatively for all *Mimiviridae* lineages) and continues to occur until the last steps of the giant virus cycle (H16 post co-infection) (Figure 2A), in contrast to the giant virus DNA replication step which seems to begin at a very early stage (described in Figure 4A for APMV). The combination of the transmission electron microscopy data and real-time PCR reports a simultaneous occurrence of the Guarani genome replication with the final stages of the giant virus morphogenesis (described in Figure 2 for APMV).

### The Host Specificity of the Guarani Virophage

**FIGURE 3:**
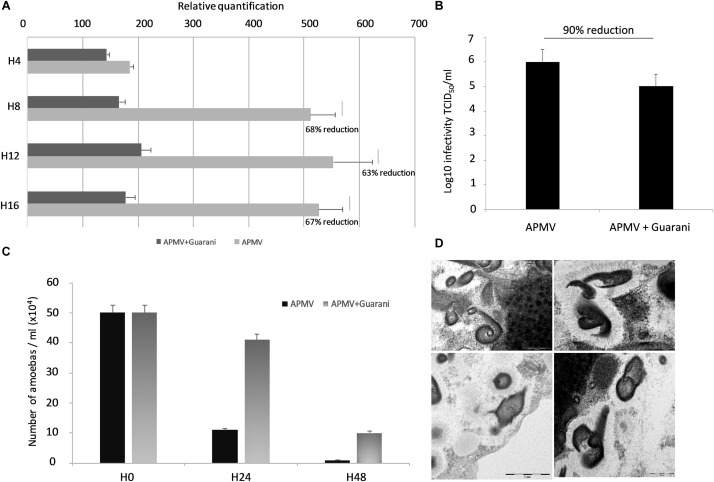
Impact of Guarani on Mimivirus cycle. **(A)** Histogram of Mimivirus growth, alone and infected with Guarani measured by Real-Time PCR from H0 to H16 post-infection. Delta Ct method was used to analyze the results as described above. **(B)** Titration of APMV infected with Guarani at 48 h post-infection compared to the control. **(C)** Quantification of the impact of Guarani on amoeba lysis. **(D)** Transmission electron microscopy images of abnormal Mimivirus particles observed in some Guarani infected cells, which were not significantly associated with the presence of virophage (scale bars 500, 200 nm, 1 μm and 500 nm, respectively).

According to the real-time PCR, Guarani replicates in the three lineages (A, B, and C) of the *Mimiviridae* family. Real-time PCR showed a 30-to-40-fold increase in Guarani DNA concentration with group A and B viruses (ΔCt = 5 approximately), versus a lower increase, 17-fold, observed with group C virus (ΔCt = 4 approximately). However, the Guarani virophage was unable to infect giant viruses that do not belong to the *Mimiviridae* family (Pandoraviruses, Cedravirus, Pithovirus, Faustovirus and Marseillevirus) (Figure 3).

### The Impact of the Guarani Virophage on Mimivirus Cycle

**FIGURE 4:**
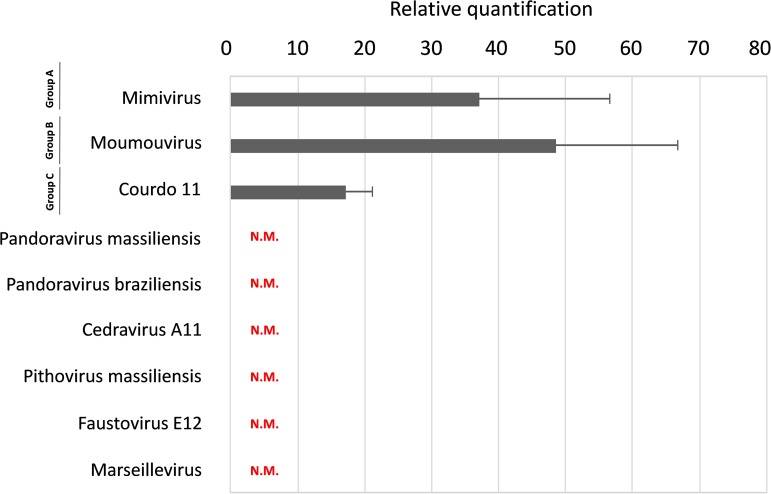
Guarani replication in different giant viruses. Histogram of Guarani replication in different *mimiviruses* belonging to group A, B, C, and other giant viruses from the putative order Megavirales. The replication was calculated by the difference in the Cycle threshold (Ct) between time points H0 and H24 measured by Real-Time PCR. The results were then analyzed with the Delta Ct method. N.M.: No multiplication.

To determine whether the infection with Guarani could affect the viral fitness of its host, we quantified the DNA replication of Mimivirus with and without Guarani virophage infection, from H0 to H16 p.i. time points. Our data suggest that APMV infected with Guarani replicates more slowly and produces significantly fewer genomic copies than Mimivirus alone during the viral cycle (Figure 4A). To quantify the virophage inhibition of Mimivirus infectivity, *A. castellanii* cells were infected with APMV (MOI = 1 TCID_50_) with or without Guarani virophage infection (MOI = 10). After 48 h of incubation, the titer of infectious particles was determined by end-point dilution, as described above. We observed that Guarani infection caused a decrease in the host virus titer (up to 90% reduction compared to the APMV which is not infected with the virophage, Figure 4B). In parallel, the number of amoebas was counted at 24 and 48 h post-infection in the presence and in the absence of Guarani virophage infection. Our results indicate that Guarani increased the host cells survival by slowing down the lysis induced by Mimivirus (Figure 4C). In a second step, we analyzed the viral factories of 200 cells co-infected with Mimivirus and Guarani using transmission electron microscopy. We highlighted the absence of a significant association between the presence of Guarani and the abnormal morphogenesis of the Mimivirus progeny (1% is the rate of abnormal particles observed both in the absence and in the presence of Guarani infection) (Figure 4D).

### The Characteristics of the Guarani Virophage Genome

**FIGURE 5:**
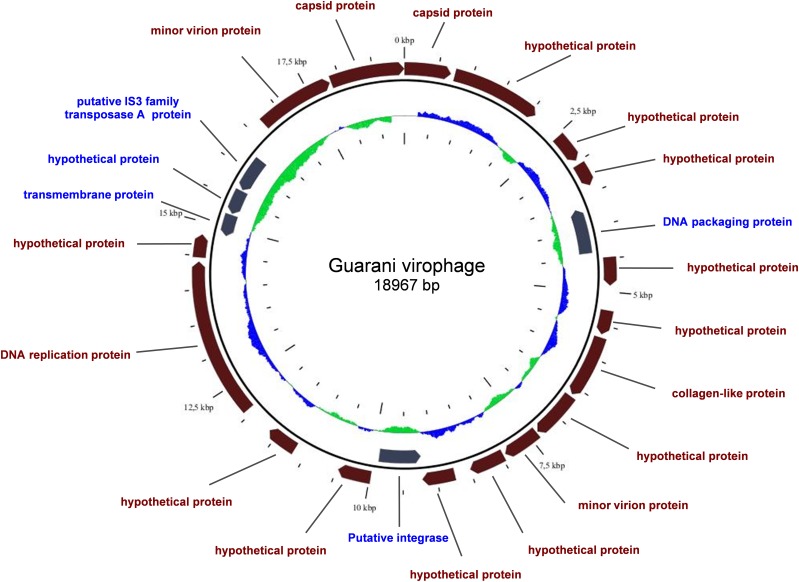
Representation of Guarani virophage genome. Predicted coding sequences on the sense strand (in brown) and the complement strand (in gray).

Guarani genome is 18,967-base-pair (bp) circular double-stranded DNA genome. The GC content of Guarani is typical of the virophages of giant viruses with a GC percentage of 26,8%. Whole nucleotide comparison with the other virophage genomes showed that Guarani is very similar to the Sputnik strains (93% coverage, 97% nucleotide identity). Similarly, Guarani has a moderate similarity with the Zamilon genome with a total coverage of 68% and a nucleotide identity of 75%. The virophage genome exhibits a high-coding capacity of about 81%. Gene prediction identified 22 ORFs (Open Reading Frame) ranging from 342 to 2340 bp in length. Most of the predicted proteins have no functional annotation (Figure 5 and Supplementary Table S1).

### Comparative Analysis With the Sputnik-Like Virophage Genomes

Eighteen different ORFs in Guarani genome displayed a very high homology in amino acids to predicted genes from Sputnik virophage (from 92 to 100%) (Table 2). Based on BLASTp and conserved domain analysis, some of these genes are predicted to encode a collagen-like protein (ORF8, ORF9), a putative integrase (ORF13), and a putative transposase (ORF20), while many of them encode unidentified proteins (ORF2, ORF3, ORF4, ORF6, ORF7, ORF14, and ORF15). Additionally, some of the identified genes encode virophage conserved core proteins. They include major capsid proteins (ORF22 and ORF1), a DNA packaging ATPase (ORF5), minor virion proteins (ORF10, ORF21), a cysteine protease (ORF11) and a DNA replication protein (ORF16).

**Table 2:** Comparison of Guarani Open Reading Frames (ORFs) with their closet homologs in Sputnik and Zamilon genomes.

Guarani ORF	Sputnik				Zamilon				Gene product
	ORF	Query cover (%)	Amino acid identity (%)	*E*-Value	ORF	Query cover (%)	Amino acid identity (%)	*E*-Value	
ORF 1	V20	100	100	7,00E-158	gp06	100	78.93	1,00E-129	Capsid protein
ORF 2	V21	100	99.77	0.0	gp07	100	70.14	0.0	Hypothetical protein
ORF 3	V1	100	98.61	1,00E-90	gp20	65	69.15	8,00E-33	Hypothetical protein
ORF 4	V2	100	98.25	4,00E-72	–	–	–	–	Hypothetical protein
ORF 5	V3	100	100	6,00E-177	gp18	100	81.22	3,00E-146	DNA packaging ATPase
ORF 6	V4	100	100	4,00E-98	gp17	98	54.55	3,00E-43	Hypothetical protein
ORF 7	V5	100	100	7,00E-74	gp16	66	60.26	9,00E-27	Hypothetical protein
ORF 8	V6	100	100	0.0	gp15	100	70.81	2,00E-134	Collagen-like protein
ORF 9	V7	100	100	3,00E-160	gp14	100	80.50	3,00E-129	Collagen-like protein
ORF 10	V8	100	98.37	2,00E-122	gp13	100	71.20	1,00E-86	Minor virion protein
ORF 11	V9	100	99.43	7,00E-123	gp12	100	76.57	3,00E-91	cysteine protease
ORF12	–	–	–	–	–	–	–	–	Hypothetical protein
ORF13	V10	100	100	1,00E-161	gp11	95	58.06	5,00E-83	Putative integrase
ORF14	V11	100	100	2,00E-107	gp10	100	52.73	3,00E-43	Hypothetical protein
ORF15	V12	100	100	4,00E-98	gp19	62	45.74	9,00E-18	Hypothetical protein
ORF16	V13	100	99.36	0.0	gp09	99	67.35	0.0	DNA replication protein
ORF17	V14	98	92.79	8,00E-53	gp08	63	52.11	4,00E-11	Hypothetical protein
ORF18	V15	51	68.33	7,00E-17	gp01	99	45.76	2,00E-07	Transmembrane protein
ORF19	–	–	–	–	gp03	96	41.22	5,00E-27	Hypothetical protein
ORF20	V17	45	96.51	4,00E-41	gp04	25	40	0.025	Putative transposase
ORF21	V18-19	99	96.36	0.0	gp05	99	66.13	1,00E-172	Minor virion protein
ORF22	V20	100	98.92	0.0	gp06	100	91.33	0.0	Capsid protein

Similarly, the Zn-ribbon domain, which is also one of the six virophage core genes, was predicted to match V14 in Sputnik genome according to a previous phylogenomic analysis (Yutin et al., 2013). We found that the ORF17 in Guarani, which is predicted to encode an unidentified protein, presents a high similarity in amino acids to V14 (98% coverage, 92% identity). On the other hand, ORF18 exhibited a moderate homology to its counterparts in Sputnik and Zamilon (V15 with 68% identity and gp01 with 45% identity, respectively). The orthologs of ORF18 in these virophages are predicted to encode a transmembrane protein which contains a putative conserved domain from cytochrome C oxydase subunit II. ORF19 did not exhibit a significant similarity to any gene in Sputnik genome, while its homolog in Zamilon (gp03 with 41% identity) encodes a protein with unknown function. The ORF20 related to putative transposase seems to be very similar to V17 in Sputnik genome (96% of amino acids identity). However, the latter presents an insertion of an adenine at the position 12936 bp of Sputnik genome. This change affects the reading frame and is associated with a reduction of the V17 length in Sputnik (88 amino acids) compared to its counterparts in Guarani and Sputnik 2 (190 and 187 amino acids, respectively). The Guarani ORF12, in contrast, did not show significant similarity to any data available in the NCBI databases. In addition, we failed to predict any conserved domain in ORF12, using CD-Search (NCBI database) and InterPro Scan (EBI database). Protein structure prediction and homology detection with HHpred (Max-Planck-Institute) and Phyr2 (Imperial College London) also failed to characterize the ORF12 product. The G+C content of ORF12 (18% on average) seems to be lower than G+C content of the rest of Guarani genome (26% on average for the complete genome, 26 and 27% on average for ORF10 and ORF13, respectively). These probably suggest that this gene has been introduced into the Guarani genome via lateral gene transfer.

**FIGURE 6:**
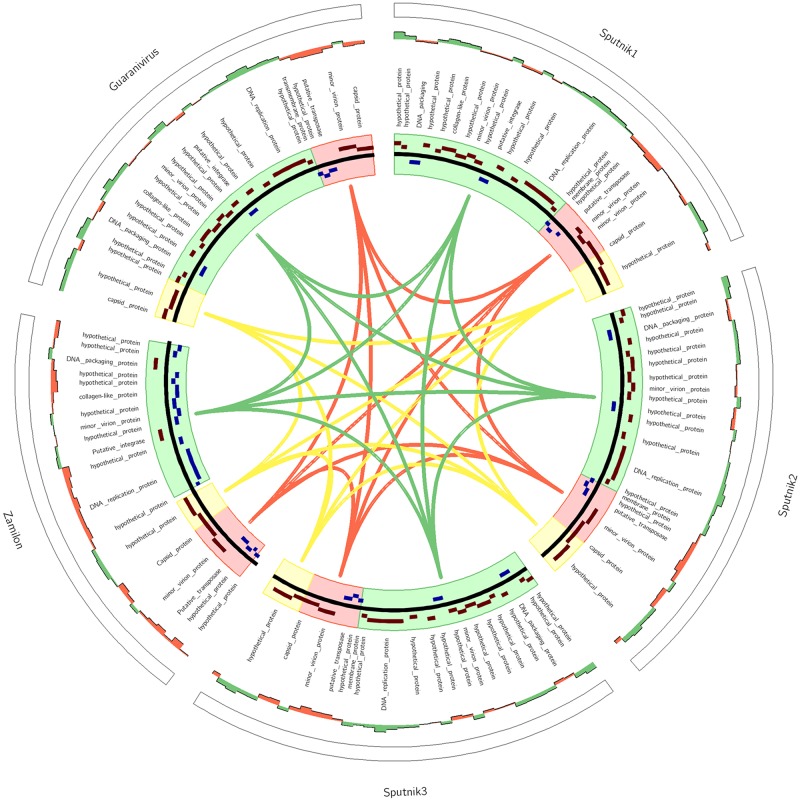
Comparison of the organization of the Guarani genome with those of other Sputnik-like virophages. The colored boxes highlight the collinear blocks of similarity between the genomes. Sense strand genes (in brown), complement strand genes (in blue).

The structure of the genome of Guarani was compared to those of the other mimivirus virophages Sputnik1, Sputnik2, Sputnik3 and Zamilon (Figure 6 and Supplementary Figure S1). Our results revealed a close architecture for all Sputnik-like virophages. This finding, along with the presence of orthologous genes between all virophages, suggests a common origin for Zamilon, Sputnik and Guarani virophages.

### Phylogenetic Analysis

We conducted a phylogenetic analysis of four conserved core proteins, Major capsid protein (MCP), minor virion protein (mCP), DNA packaging ATPase and putative cysteine protease homolog in Guarani genome, in addition to ORF15, which is related to counterpart genes found in mimiviruses genomes. As expected, the phylogenetic tree, based on MCP, revealed that Guarani virophage is most closely related to Sputnik strains. Phylogenies for minor virion protein (ORF21), DNA packaging ATPase and putative cysteine protease strongly support that Guarani is clustered within the Sputnik strains group. The phylogenetic reconstruction of the ORF15 showed a topology similar to that observed in previous analyses (Gaia et al., 2014; Bekliz et al., 2015), with this gene being clustered with Sputnik strains closer to group A *Mimiviridae*, while Zamilon and Zamilon 2 were clustered with group B and C *Mimiviridae* lineages (Supplementary Figure S2).

### Search for Mimivirus Late Promoter in the Guarani Genome

**FIGURE 7:**
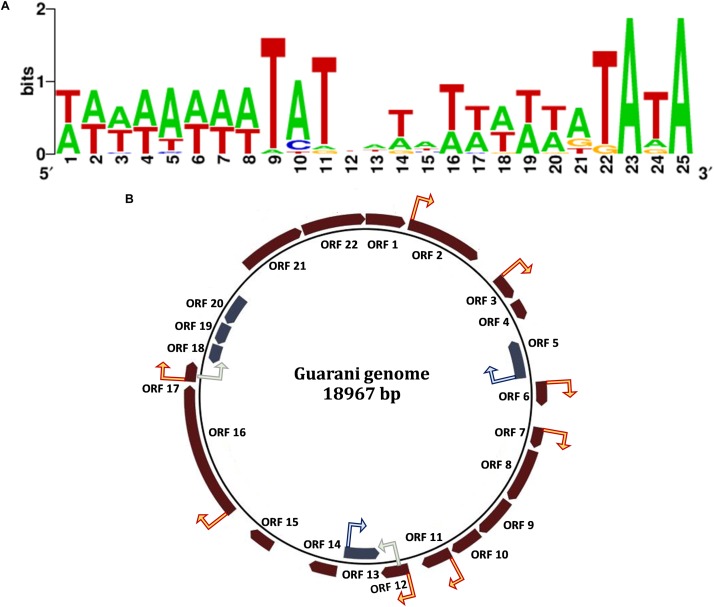
The Mimivirus late promoter found in Guarani genome. **(A)** The promoter motif sequence logo. **(B)** The Mimivirus late promoter distribution in Guarani genome, red arrows (promoters in upstream of genes found in the positive strand), blue arrows (promoters in upstream of negative strand genes), gray (promoter motifs in coding region).

It is known from previous studies that the Mimivirus late promoter was present in Sputnik genome, suggesting that the virophage genes are expressed by the Mimivirus transcription complex (Poirot et al., 2010). We therefore screened the upstream regions of all Guarani genes for the presence of the Mimivirus late promoter motif and found that this motif was associated with 10 Guarani genes confirming what has been described for Sputnik (Figure 7).

## Discussion

So far, giant viruses have been shown to be host for seven isolated strains of virophages that depend on and parasitize the viral factories of a wide range of *Mimiviridae* members (La Scola et al., 2008; Fischer and Suttle, 2011; Desnues et al., 2012; Gaia et al., 2013, 2014; Campos et al., 2014; Michel et al., 2018). Through this study, we report the isolation and characterization of the eighth virophage that we named Guarani. Like Sputnik 3, the new virophage was isolated without detectable giant viruses, probably because the amoeba strains used here were not susceptible to the original host virus, and that the latter is not targeted by our molecular mimivirus screening system. Guarani is closely related to the other virophages, Sputnik and Zamilon, already described in previous studies. Guarani has the same morphological characteristics as the Sputnik, Zamilon and Mavirus strains, with icosahedral particles 50 to 60 nm in diameter (La Scola et al., 2008; Fischer and Suttle, 2011; Gaia et al., 2014).

In terms of genetic content, Guarani has 18 kb circular double-stranded DNA genome and contains 22 putative ORFs. The size and the organization of its genome are typically analogous to the other known virophage genomes. Sputnik is the closest from Guarani with 18 kbp and 21 predicted ORFs (18 kbp and 20 ORFs for Sputnik2, Sputnik3, and Rio Negro virophage) (La Scola et al., 2008; Desnues et al., 2012; Gaia et al., 2013; Borges et al., 2018). In addition, most predicted Guarani proteins had a high homology to predicted Sputnik proteins. These proteins comprise the core conserved genes of virophages, such as capsid proteins and proteins involved in the DNA viral replication. Interestingly, the main novelty of Guarani compared to the other Sputnik strains genomes is ORF12. The absence of significant similarity between this gene and genome sequences of other known viruses indicates probably that this gene has been introduced in Guarani genome via lateral gene exchange. ORF19 also distinguishes Guarani from Sputnik, but a distant homolog of this gene is present in Zamilon genome.

On the other hand, Zamilon genome with its 17 kbp and 20 ORFs also exhibited moderate to high homologies to predicted Guarani virophage proteins (Gaia et al., 2014). The size of the Guarani genome and the number of ORFs are also similar to Mavirus which contains 19 kbp with 20 ORFs (Fischer and Suttle, 2011). Similarly, Phaeocystis globosa virophage and the other non-isolated virophages discovered by metagenomics present comparable genetic features, their genome sizes range from 17–29 kbp and encode from 21–34 predicted ORFs (Bekliz et al., 2016).

Mimiviruses particles apparently package a complete transcription apparatus used at the early stage of infection to initiate their own gene transcription, notably genes coding for genome replication machinery (Raoult et al., 2004; Renesto et al., 2006; Fischer et al., 2010). In contrast, virophages depend on the transcription system of their giant host viruses, as demonstrated by specific promoters and polyadenylation signals shared between virophages and their host giant viruses. Conserved AT-rich promoters, associated with a late expression, were found upstream of 12 Sputnik coding sequences, suggesting that Sputnik expression has a late occurrence in Mimivirus factory under the control of Mimivirus machinery (Claverie and Abergel, 2009; Poirot et al., 2010; Desnues and Raoult, 2012). In this paper, we strongly confirmed that unlike the replication of the giant virus genome which occurs at the early stage of co-infection, the replication of Guarani virophage genome begins at a later stage and extends until the final stages of Mimivirus morphogenesis. Additionally, transmission electronic microscopy failed to detect mature virophage particles before the final stage of the viral cycle, a morphologic feature clearly different to that observed previously with Sputnik and Zamilon virophages. These results were reinforced by the detection of the Mimivirus late promoter in the upstream regions of 10 Guarani genes. These genes include those putatively encoding for DNA replication and DNA packaging proteins (Figure 7). Taken together, our results, along with previous findings, support the model of the parasitic pathway, adopted by virophages exploiting the genome replication apparatus of their giant host virus, to replicate their own genomic material.

According to our results, Guarani virophage appears to negatively impact the replication efficiency and the infectivity of its host mimivirus (68 and 90% decrease, respectively). Additionally, we observed that this virophage is able to decrease the lytic capacity of the host virus. Comparable results were obtained with Sputnik and Sputnik 3. It has been shown that the latter induces a decrease of up to 75% in the fitness of replication of APMV (Gaia et al., 2013). Similarly, RNV has been implicated in a drastic decrease in APMV infectivity (up to 99.9%), leading to an increase in the survival of the host cell population (9). Mavirus also decreased the infectivity of its host virus up to more than 99% (from 10^9^ to less than 10^7^ TCID50/ml) compared to the 90% reduction observed for Guarani (Fischer and Hackl, 2016). In addition, it was reported that all these virophages were associated with a significant increase in the production of abnormal particles of their mimivirus hosts. It is striking that this harmful effect on the host virus morphogenesis was not detected with Guarani virophage infection. Zamilon, on the other hand, does not appear to be associated with any deleterious effects on the viral replication, the infectivity or the morphogenesis of the host giant virus (Gaia et al., 2014).

The negative effect of Guarani on its host virus is lower than what has been observed for some virophages such as Mavirus. However, in most cases, the presence of virophages seems to play a potential role in the giant virus-host cell interaction. By reducing the production and the virulence of giant viruses, these small predators probably regulate the stability of the microbial population (amoebae and marine protists). This deduction corroborates with the conclusion of a metagenomic study performed on samples from the Organic Lake, a hypersaline meromictic lake in Antarctica. According to this study, the introduction of virophages in this ecosystem stimulates the growth of its microbial web by decreasing the mortality of the phototrophic algae induced by their invader giant viruses (Yau et al., 2011). In addition to this ecological study, a mathematical model was applied to study the dynamic interactions between host populations, their giant viruses and virophages. In this model, the virophages confer not only a direct protective role for the host amoeba by attacking their giant viruses, but the presence of virophages could deeply alter the evolutionary course of the giant virus population by selecting viral clones with weaker reproductive ratio. Consequently, the model suggested that the dynamic instabilities caused by this change could make virophage population more susceptible to extinction (Wodarz, 2013). As described above, a relevant feature of Guarani virophage is its late replication cycle which allows probably its host mimivirus to replicate and produce some progenies. We speculate that this feature helps in maintaining a population of giant viruses in the environment and thus promotes the expansion of virophages. Nevertheless, this feature was investigated for Guarani only, further experiments are thus needed to confirm it for the other virophages.

Guarani, like all Sputnik strains, has the capacity to infect the three lineages of the *Mimiviridae* family (Gaia et al., 2013). This feature, coupled with other evidence of viral signatures in the Sputnik genome, stimulates debate about the role of virophages as potential vectors of gene transfers between mimiviruses (Desnues and Raoult, 2012). Interestingly, all virophages described so far were isolated and cultivated exclusively with giant viruses from the *Mimiviridae* family, which are themselves relatively similar to each other. In this study, Guarani was tested against Pandoraviruses (Pandoravirus massiliensis and Pandoravirus braziliensis) (Aherfi et al., 2018), Cedravirus A11 (Andreani et al., 2016), Pithovirus massiliensis (Levasseur et al., 2016a), Faustovirus E12 (Rossmann et al., 2015) and Marseillevirus (Yutin et al., 2009). Our data revealed that the virophage is not able to infect viruses from these groups, probably because of their phylogenetic distance from mimiviruses. Their transcription apparatus may not be adapted for the virophage genes, but more analysis is required to confirm this. Similarly, Sputnik was previously tested against Marseillevirus and exhibited similar results (Raoult, 2010). Further studies are nevertheless required to explore virophages specificity against other Megavirales members. On the other hand, Zamilon is the unique virophage isolated so far that can grow on the lineage B and C, but not on the lineage A *Mimiviridae* (Gaia et al., 2014). This host specificity of Zamilon led us to discover the MIMIVIRE system, which represents a nucleic-acid-based immunity, conferring resistance to lineage A mimiviruses against Zamilon virophage infection. This resistance seems to be acquired through the incorporation of short repeated sequences from Zamilon genome (ORF4) in the R349 gene of Mimivirus. A putative Cas-like sequence was identified near the R349 and linked to the interference mechanism (Levasseur et al., 2016b; Dou et al., 2018). Therefore, we screened the R349 gene of Mimivirus as well as its orthologs in Moumouvirus and Courdo-11 for the presence of probable Guarani repeated sequences. Our searches confirmed the absence of repeated sequences that were homologous to Guarani in the analyzed genes, thus corroborating the results of the multiplication tests.

The new Guarani virophage is consistent with the conventional definition of a virophage as a parasite of giant viruses, exploiting the viral host machinery for genome replication and transcription and impairing the normal replicative cycle of its host. In addition, like Sputnik, Guarani has a wide spectrum of replication through its ability to infect all groups of *Mimiviridae*. This feature may allow the virophage to drive the horizontal exchange of genes between giant viruses of the family *Mimiviridae*, thus contributing to their genomic diversity. Our results show that Sputnik, Zamilon, Mavirus and RNV are not the unique virophages of giant viruses and suggest that these small biological entities are more complex than what was previously thought.

## Author Contributions

SM performed experiments, analyzed the results, and wrote the manuscript. MB isolated the virus and performed preliminary identification. JA conceived the study. FP performed electronic microscopy experiments. AL analyzed genomic data. BS conceived the study and wrote the manuscript.

## Conflict of Interest Statement

The authors declare that the research was conducted in the absence of any commercial or financial relationships that could be construed as a potential conflict of interest.

## Footnotes

1 http://weblogo.berkeley.edu

## Supplementary Material

FIGURE S1 Linear genome alignment of Guarani, Sputnik, Sputnik2, Sputnik3, and Zamilon, the boxes highlight the collinear blocks of similarity between the genomes the *y* axes of boxes represent the average similarity of each region. This schematic genome alignment diagram was obtained using the Mauve software package (Darling et al., 2004).

FIGURE S2 Phylogenetic reconstructions using amino acid sequences of major and minor capsid proteins **(A,B)**, DNA packaging protein **(C)**, cysteine protease-like **(D)** and ORF15 **(E)**, of Guarani with their homologs found in the other virophage genomes. The analysis was performed using MEGA version 7.0, applying the maximum-likelihood method and WAG model of evolution with 500 bootstrap replicates (cutoff ≥ 50).

TABLE S1 Guarani genome functional annotation.
