# Supplementary figures and images for: Guarani Virophage, a New Sputnik-Like Isolate From a Brazilian Lake
Source: Front Microbiol. 2019 May 3;10:1003. doi: 10.3389/fmicb.2019.01003 (PMC6510173; doi:10.3389/fmicb.2019.01003)

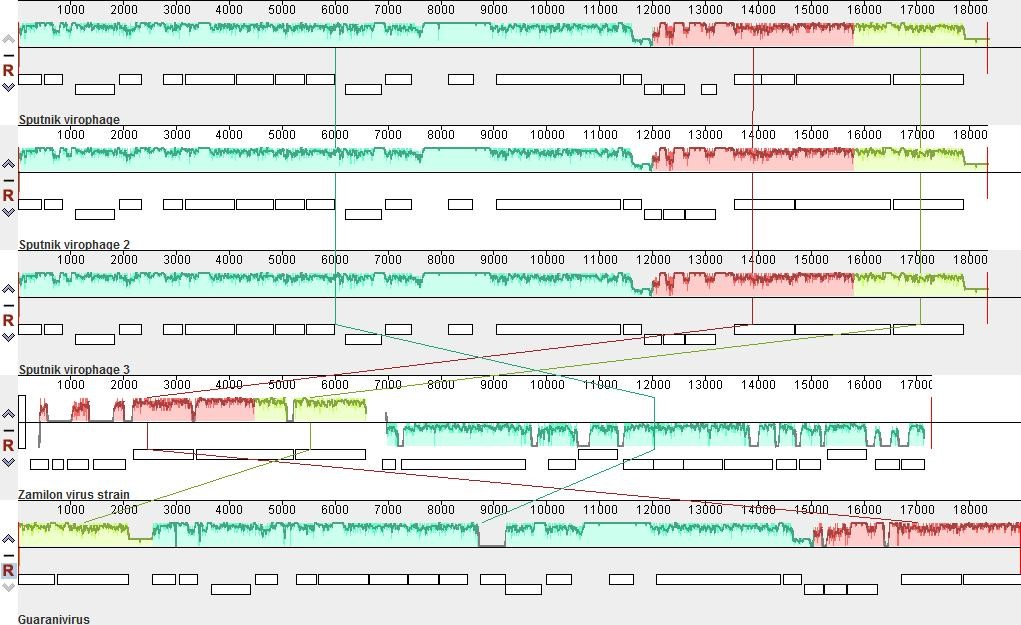

Supplement: FIGURE S1 — Linear genome alignment of Guarani, Sputnik, Sputnik2, Sputnik3, and Zamilon, the boxes highlight the collinear blocks of similarity between the genomes the y axes of boxes represent the average similarity of each region. This schematic genome alignment diagram was obtained using the Mauve software package (Darling et al., 2004). [file Image_1.TIF]

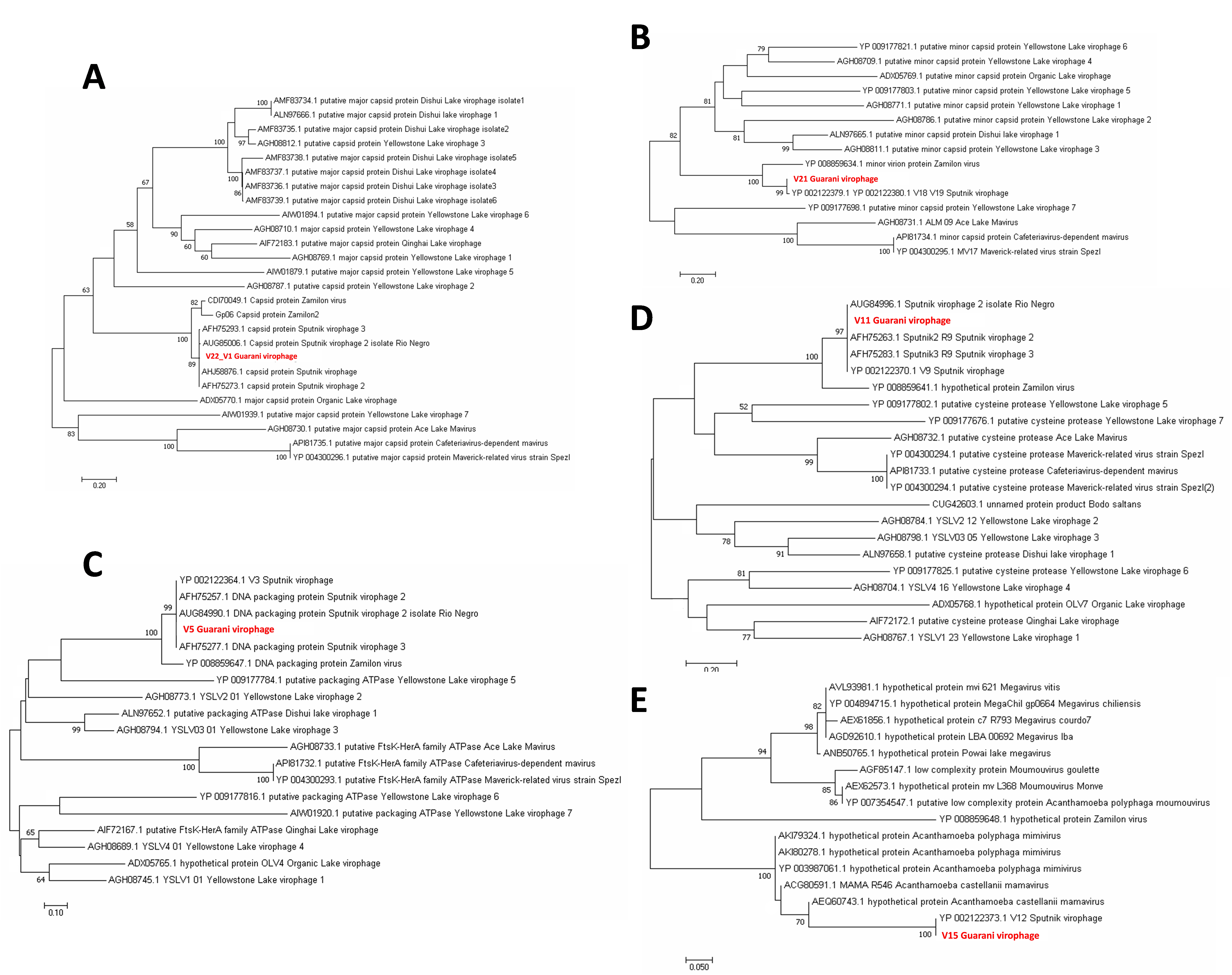

Supplement: FIGURE S2 — Phylogenetic reconstructions using amino acid sequences of major and minor capsid proteins (A,B), DNA packaging protein (C), cysteine protease-like (D) and ORF15 (E), of Guarani with their homologs found in the other virophage genomes. The analysis was performed using MEGA version 7.0, applying the maximum-likelihood method and WAG model of evolution with 500 bootstrap replicates (cutoff ≥ 50). [file Image_2.TIF]
